# Designing Advanced Electrolytes for High‐Voltage High‐Capacity Disordered Rocksalt Cathodes

**DOI:** 10.1002/smll.202501600

**Published:** 2025-03-24

**Authors:** Ridwan A. Ahmed, Rohith Srinivaas Mohanakrishnan, Jingyang Wang, Krishna P. Koirala, Qian Zhao, Yanbao Fu, Ying Chen, Justin C. Rastinejad, Tianyu Li, Lirong Zhong, Mateusz Zuba, Carrie Siu, Ozgenur Kahvecioglu, Raphaële J. Clément, Bryan D. McCloskey, Vincent S. Battaglia, Kristin Persson, Chongmin Wang, Wu Xu

**Affiliations:** ^1^ Energy and Environment Directorate Pacific Northwest National Laboratory Richland WA 99354 USA; ^2^ Department of Materials Science and Engineering University of California Berkeley Berkeley CA 94720 USA; ^3^ Physical and Computational Sciences Directorate Pacific Northwest National Laboratory Richland Richland WA 99354 USA; ^4^ Environmental Molecular Sciences Laboratory Pacific Northwest National Laboratory Richland Richland WA 99354 USA; ^5^ Energy Storage and Distributed Resources Division Lawrence Berkeley National Laboratory Berkeley CA 94720 USA; ^6^ Department of Chemical and Biomolecular Engineering University of California Berkeley Berkeley CA 94720 USA; ^7^ Materials Department and Materials Research Laboratory University of California Santa Barbara Santa Barbara CA 93106 USA; ^8^ Applied Materials Division, Argonne National Laboratory Lemont IL 60439 USA; ^9^ Department of Materials Science and Engineering Berkeley CA 94720 USA; ^10^ Division of Materials Science Lawrence Berkeley National Laboratory Berkeley CA 94720 USA

**Keywords:** cathode electrolyte interphase, disordered rocksalt cathode, electrolyte, high voltage, solid electrolyte interphase

## Abstract

Lithium (Li)‐excess transition metal oxide materials which crystallize in the cation‐disordered rock salt (DRX) structure are promising cathodes for realizing low‐cost, high‐energy‐density Li batteries. However, the state‐of‐the‐art electrolytes for Li‐ion batteries cannot meet the high‐voltage stability requirement for high‐voltage DRX cathodes, thus new electrolytes are urgently demanded. It has been reported that the solvation structures and properties of the electrolytes critically influence the performance and stability of the batteries. In this study, the structure–property relationships of various electrolytes with different solvent‐to‐diluent ratios are systematically investigated through a combination of theoretical calculations and experimental tests and analyses. This approach guides the development of electrolytes with unique solvation structures and characteristics, exhibiting high voltage stability, and enhancing the formation of stable electrode/electrolyte interphases. These electrolytes enable the realization of Li||Li_1.094_Mn_0.676_Ti_0.228_O_2_ (LMTO) DRX cells with improved performance compared to the conventional electrolyte. Specifically, Li||LMTO cells with the optimized advanced controlled‐solvation electrolyte deliver higher specific capacity and longer cycle life compared to cells with the conventional electrolyte. Additionally, the investigation into the structure–property relationship provides a foundational basis for designing advanced electrolytes, which are crucial for the stable cycling of emerging high‐voltage cathodes.

## Graphical abstract

Lithium batteries with high energy density are critical for advancing electric transportation and grid storage. By integrating simulations and experiments, advanced electrolytes with controlled solvation structures are developed. These electrolytes form ultrathin, stable, and robust protective interphases on high‐voltage, high‐capacity disordered rocksalt cathodes, preserving structural integrity, and enhancing the performance of lithium batteries with such cost‐effective and high‐energy‐density cathodes.

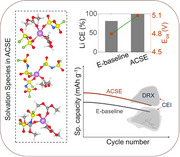

## 1 Introduction

The development of low‐cost and high‐energy‐density lithium (Li) batteries is essential to address the global energy storage demands. Although significant advancements have been achieved in the anode component of Li batteries, particularly with the utilization of Li metal anode with high specific capacity,^[^ ^1^ ^]^ the cathode remains a major limiting factor. Additionally, the state‐of‐the‐art cathodes predominantly rely on expensive non‐abundant transition metals like cobalt (Co) and nickel (Ni) hindering cost reduction and having sustainability issues.^[^ ^2^ ^]^ Therefore, the development of inexpensive high‐capacity cathode materials is critical for the realization of low‐cost and high‐energy‐density Li‐ion and Li metal batteries. Among the emerging high‐capacity cathode materials, Li‐excess, inexpensive transition metal oxide materials which crystallize in disordered rock salt (DRX) structure seem particularly promising as they offer both high capacity and cost benefits.^[^ ^3^ ^]^ Additionally, they are typically made from earth‐abundant materials such as manganese (Mn) with high thermal stability.^[^ ^4^ ^]^ However, these cathodes require high voltage cut‐offs (≈ 4.8 V) to unlock their high‐capacity potential. Thus, cycling these cathodes in conventional LiPF_6_/organocarbonate‐based electrolytes with low oxidation potential (≈ 4.3 V) introduces several challenges. These include the formation of unstable cathode electrolyte interphase (CEI), parasitic side reactions between the cathode and the electrolyte, and electrolyte decomposition leading to significant gas evolution during cycling.^[^ ^5^ ^]^ Furthermore, DRX cathodes face inherent challenges such as bulk structural instability and oxygen (O) release from the irreversible anionic redox processes which further exacerbate parasitic reactions with the electrolyte.^[^ ^6^ ^]^ These issues collectively result in poor electrochemical cycling performance of the DRX cathodes.^[^ ^7^ ^]^ Therefore, advanced electrolytes with unique solvation structures and characteristics which allow for the mitigation of these challenges are necessary to enable high capacity and improved cycling performance of these high‐voltage cathodes.

Several electrolytes have been proposed including ionic liquid electrolytes,^[^ ^5^, ^8^ ^]^ and highly concentrated electrolytes (HCEs),^[^ ^9^ ^]^ despite achieving certain successes with these electrolytes, the cycling performance of these cathodes is still poor. Additionally, HCEs have inherent problems such as high viscosity, poor wettability, poor ionic transport properties, and high cost. Recently, we demonstrated the use of a localized high‐concentration electrolyte (LHCE) with an ethylene carbonate (EC) additive enabled an improved performance of Li_1.13_Mn_0.66_Ti_0.21_O_2_ (LMTO) DRX cathode and offered several advantages over other electrolytes which were previously reported for the DRX cathodes.^[^ ^10^ ^]^ This underscores the effectiveness of the LHCE for enabling stable cycling of LMTO DRX cathode and mitigating several issues associated with them. Moving forward, understanding the effects of the electrolyte composition and solvation structure on the electrolyte properties and the cycling performance of the DRX cathodes is critical to inform the development of next‐generation electrolytes for these cathodes.

In this work, we report the design of appropriate electrolytes for high‐voltage DRX cathodes by using the combination of theoretical calculations and experiments and investigating the effects of solvation structures and properties of a series of electrolytes based on Li bis(fluorosulfonyl)imide (LiFSI) salt, dimethyl carbonate (DMC) solvent and 1,1,2,2‐tetrafluoroethyl‐2,2,3,3‐tetrafluoropropyl ether (TTE) diluent on the cycling stability of a DRX cathode Li_1.094_Mn_0.676_Ti_0.228_O_2_ (LMTO) whose characterization data are shown in Figures S1,S2 and Table S1 (Supporting Information). By varying the composition of solvent and diluent, advanced electrolytes with unique structures and characteristics are achieved, and the optimized electrolyte with a controlled‐solvation structure exhibits significantly reduced amount of free solvent molecules and the formation of large aggregates, resulting in enhanced high‐voltage stability and long‐term cycling performance in Li||DRX cells, superior to the conventional electrolyte of 1 m LiPF_6_ in EC‐DMC (termed as E‐baseline).

## 2 Results and Discussion

### 2.1 Electrolyte Design, Structures and Properties

The solvation structures of electrolytes, a critical determining factor of the cycling performance of Li‐ion and Li metal batteries, can be precisely tuned through compositional modifications. For instance, the outstanding performance of LHCEs is due to their unique solvation structures, which differ markedly from those of conventional electrolytes.^[^ ^11^ ^]^ To develop advanced electrolytes with good compatibility with LMTO DRX cathode, we designed and investigated a series of electrolytes based on LiFSI salt, DMC solvent, and TTE diluent, systematically varying the solvent‐to‐diluent ratios. The electrolyte formula is written in LiFSI‐*x*DMC‐*y*TTE (by mol), where *x* = 1.6, 2, 3, 4, and 5 while *y* = 1, 2 and 3, respectively.

**Figure 1:**
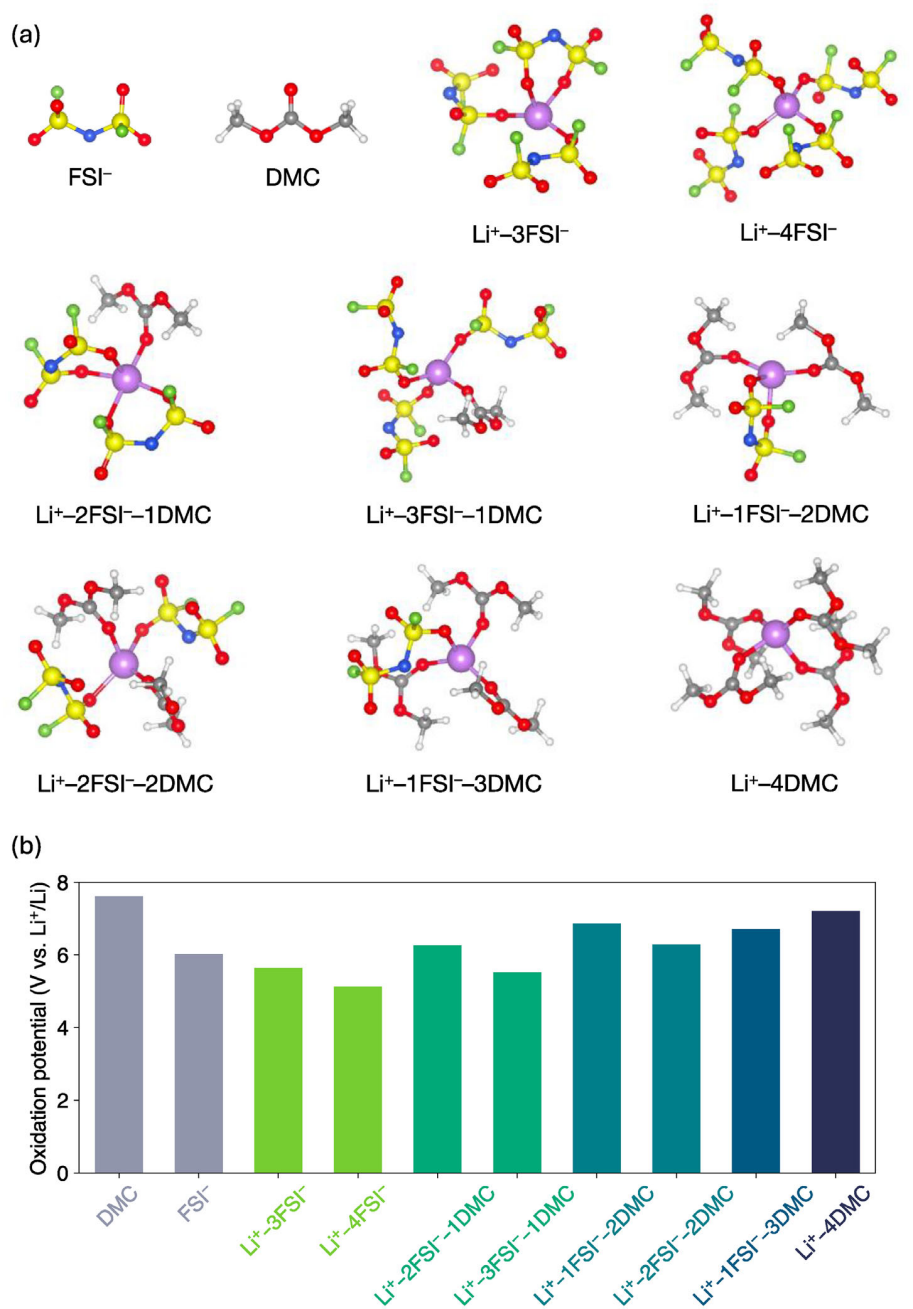
a) Structures and b) oxidation potentials of the primary Li^+^ solvation species identified in studied electrolytes from MD simulations and DFT calculations, respectively.

The structures of the Li^+^ solvation species in the studied electrolytes were investigated via molecular dynamics (MD) simulations and are presented in **Figure** **1**. The solvation shell speciation statistics are summarized in Figures S3–S7 (Supporting Information). It is readily seen from Figures S3–S7 (Supporting Information) that the TTE molecules do not populate the first solvation shells of Li^+^; specifically, the TTEs present beyond 4 Å from Li^+^; furthermore, it is evident that Li^+^ prefers a four‐fold coordination in LHCEs, which agrees with previous reports.^[^ ^12^ ^]^ Most notably, the solvation shell speciation statistics show a strong dependence on the overall relative composition of solvents and salts. As shown in Figures S3–S7 (Supporting Information), in the LiFSI‐*x*DMC‐*y*TTE solutions, the FSI^–^/DMC ratio in the most prevalent solvation shell composition gradually decreases from 2:2 for *x* = 1.6 and 2, to 1:3 for *x* = 3 and 4, to 0:4 for *x* = 5. More generally, solvation shells with higher DMC content become more prevalent as *x* increases. In addition, as the overall relative DMC concentration increases, the distribution of Li^+^ solvation shell compositions becomes more uneven. Specifically, the two most populated solvation shell compositions account for more than 95% of all the existing solvation shell compositions for *x* = 5, compared with only 60%‐70% for *x* = 1.6. Interestingly, we found that the overall relative concentration of TTE can also affect the population statistics of first solvation shell compositions, albeit to a lesser extent compared with the solvent and salt concentrations. The impact of TTE on the solvation shell statistics is most pronounced at a high DMC concentration (*x* = 4 and 5), where the populations of 1FSI^−^–3DMC increase relative to those of 0FSI^−^–4DMC.

Furthermore, electrolytes with high concentrations of LiFSI exhibit contact ion pairs (CIPs) as the primary solvation shells (Figures 1 and Figures S3–S7, Supporting Information). Based on the Li^+^ solvation structures, the oxidation potentials (E_ox_) were computed using density functional theory (DFT) and are shown in Figure 1b. We observe that Li^+^‐coordination generally lowers the oxidation potential compared with those for the uncoordinated individual solvent and salt molecules. In particular, the oxidation potentials are lower for solvation complexes with a higher concentration of FSI^–^, which agrees with expectations from electrostatic considerations. In all the cases, the E_ox_ values show that the primary solvation structures are stable up to at least 5 V versus Li^+^/Li and hence are stable against the high‐voltage DRX cathodes. It should be noted that these calculated oxidation potentials are for the selected solvation species but not for the electrolytes.

To save time and effort in simulations and calculations, the electrochemical performance of the fifteen electrolytes was screened by preliminarily cycling them in Li||LMTO coin cells in the voltage range of 2.0‐4.8 V. As shown in Figure S8 (Supporting Information), the electrolytes with higher DMC content and lower TTE content do not perform well, thus only 3TTE electrolytes (i.e., LiFSI‐*x*DMC‐3TTE, *x* = 1.6, 2, 3, 4, 5) and 1.6DMC electrolytes (i.e., LiFSI‐1.6DMC‐*y*TTE, *y* = 1, 2, 3), totally seven, were selected for further investigation.

**Figure 2:**
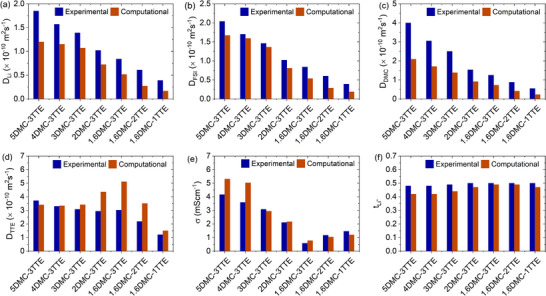
Comparison of transport properties of the studied electrolytes from MD simulations and experimental measurements. a–d) Diffusion coefficients of Li^+^ a), FSI^−^ b), DMC c), and TTE d,e) Room‐temperature (25 °C) ionic conductivity f) Li^+^ transference number.

The ionic transport properties such as ionic conductivity and diffusion coefficients of Li^+^, FSI^−^, DMC, and TTE of the seven electrolytes were computed using MD simulations and the obtained results are summarized in Table S2 (Supporting Information). On the other hand, the actual room‐temperature ionic conductivity and temperature‐dependence of viscosity of the selected electrolytes were measured by electrochemical impedance spectroscopy (EIS) and viscometer, and the corresponding curves are shown in Figure S9a,b (Supporting Information), respectively. Meanwhile, the diffusion coefficients of the four species were measured by pulsed‐field gradient nuclear magnetic resonance (PFG‐NMR) spectroscopy under a non‐electric field. All results are listed in Table S3 (Supporting Information). Moreover, the Li^+^ transference numbers in these electrolytes were calculated using the equation of *D*_+_/(*D*_+_ + *D*_‐_) from the computation and PFG‐NMR results, respectively, and summarized in Tables S2,S3 (Supporting Information), respectively. To have an easy comparison, the simulation and experimental results of ionic conductivity, diffusion coefficient, and Li^+^ transference number are also plotted in **Figure** **2**. The computed diffusion coefficients for all the species, conductivities, and ideal transference numbers are qualitatively in good agreement with the experimental results. It is observed that with the decrease of DMC amount in the electrolytes or the DMC‐TTE mixtures from 5 to 1.6, the diffusion coefficient of Li^+^ and the ionic conductivity of the electrolytes decrease as shown in Figure 2. Additionally, the electrolytes with the lowest DMC amount (1.6DMC–*y*TTE, *y* = 1, 2, 3) show the lowest salt dissociation degree which increases with decreasing the amount of TTE. The decrease in ionic conductivity is accompanied with an increase in viscosity as the DMC content is reduced from 5 to 1.6 in the DMC‐TTE mixtures. However, the dissociation degree as well as the ionic conductivity increase as the TTE content varies from 1 to 3 in the 1.6DMC electrolytes despite of the increase in viscosity. This suggests that the ionic conductivity of the low DMC electrolytes is largely governed by the salt dissociation degree but not the viscosity of the electrolyte. This finding also confirms that the amount of diluent (TTE) can affect the dissociation degree and the overall transport properties of the LHCEs as observed in prior studies.^[^ ^13^ ^]^ In addition, the electrolytes with low amounts of DMC (2 and 1.6) exhibit a Li^+^ transference number of ≈ 0.5 based on self‐diffusion coefficient values of the cation and anion measured under a non‐electric field condition, which is slightly higher than for the conventional electrolyte, 1 m LiPF_6_ in EC‐DMC (1:2 by wt.) (E‐baseline) (0.42, Table S3, Supporting Information).

Besides the transport properties, the oxidation potential for each electrolyte was measured on the conductive carbon‐coated aluminum (C@Al) foil working electrode using the linear sweep voltammetry (LSV) method and the results are shown in Figure S9c and Table S3 (Supporting Information). The results show the electrolytes with 3–5DMC in the solvent mixtures have the oxidation potentials of only ≈4.3 V versus Li/Li^+^, much lower than the baseline electrolyte (≈4.8 V). Decreasing DMC content to 2 can increase the oxidation potential to ≈4.5 V, and further decreasing DMC to 1.6 enables the electrolytes to show a superhigh oxidation potential of ≈5.1 V, outperforming the E‐baseline. The difference in values of the computed oxidation potentials from the solvation structures and the experimental values is because the active conductive carbon electrode with high surface area and catalytic effect will greatly reduce the oxidation potential of an electrolyte when comparing the flat and inert electrodes like platinum, glassy carbon, and Al, especially if there are more free solvent molecules and solvent‐separated ion pairs in 3–5DMC based electrolytes. The extremely low content of the solvating solvent in the electrolyte ensures very limited free solvent molecules and then achieves high oxidation stability.

In addition, the cathodic stability of the electrolytes was evaluated by the average Li Coulombic efficiency (CE) of the various electrolytes measured in Li||copper (Cu) cells. As shown in Figure S10 (Supporting Information), increasing DMC content in the solvent mixture reduces the average Li CE, meaning poor cathodic stability. With 2 and 1.6 DMC, the electrolytes show high values of CE over 99%. This is largely attributed to their unique localized high‐concentration solvation structures allowing for a more uniform Li deposition and formation of a stable interphase layer. However, the electrolytes with DMC > 3 show low CE, which is because the high amount of DMC in these electrolytes results in a poor and unstable interphase layer.^[^ ^14^ ^]^

Following the above results, the electrolyte of LiFSI‐1.6DMC‐3TTE (by mol) with high oxidation potential (≈5.1 V) and high Li CE (99.6%), termed as advanced controlled‐solvation electrolyte (ACSE), was selected for more studies in electrochemical performance and gassing on LMTO cathode under high voltage, in comparison with E‐baseline.

### 2.2 Electrochemical Cycling Performance of Li||LMTO Cells

**Figure 3:**
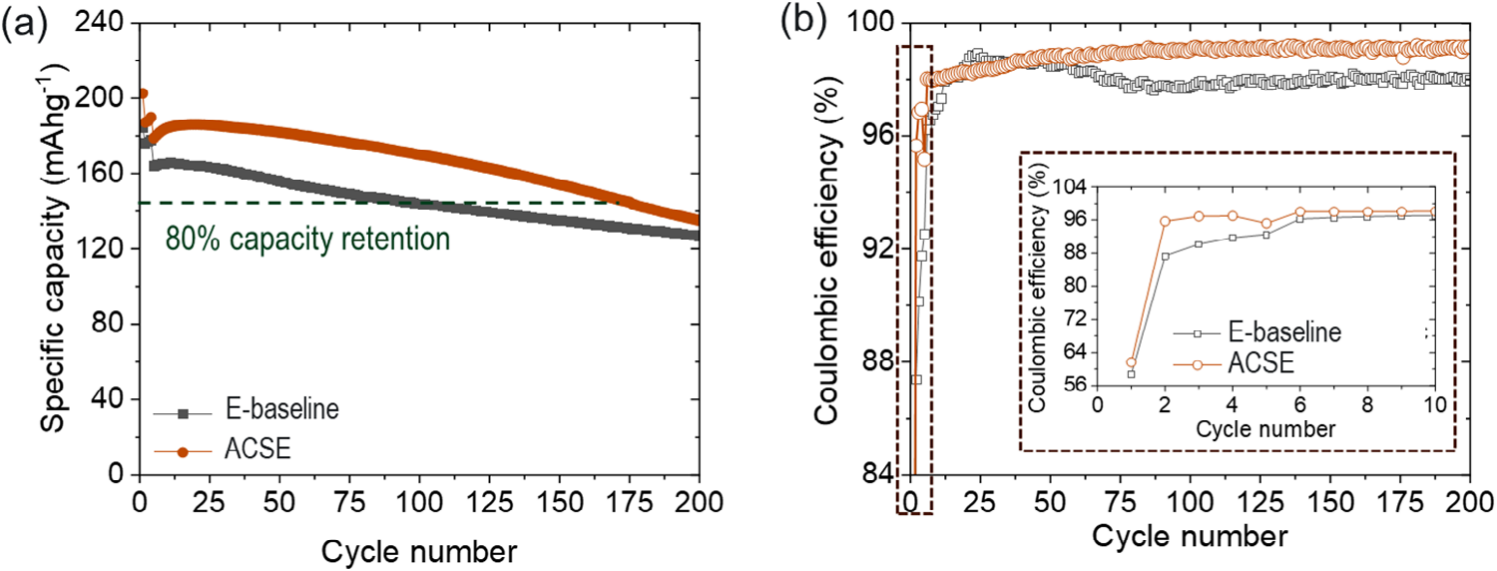
Extended cycling performance of Li||LMTO cells using the ACSE and E‐baseline at 20 mA g^−1^ charge/discharge rate, after four formation cycles at 10 mA g^−1^, in the voltage range of 2.0−4.8 V. a) Discharge specific capacity, b) Coulombic efficiency.

The electrochemical performance of the ACSE and E‐baseline was evaluated in Li||LMTO coin cells to investigate their potential for enabling Li||LMTO cells with improved performance at the high charge cutoff voltage of 4.8 V. The cells were subjected to four initial formation cycles at a charge/discharge current density of 10 mA g^−1^ and then cycling at a current density of 20 mA g^−1^ for both charge and discharge processes. The results of the extended cycling for Li||LMTO cells with ACSE and E‐baseline are presented in **Figure** **3** and the charge/discharge voltage profiles at selected cycle numbers are shown in Figure S11 (Supporting Information).

Figure S11 (Supporting Information) reveals the lower voltage polarization for cells with ACSE than for cells with E‐baseline, which indicates lower impedance buildup in Li||LMTO cells with the ACSE. At the 1^st^ formation cycle at 10 mA g^−1^, the cells with ACSE deliver a specific capacity of 202.6 mAh g^−1^ and a first cycle CE (FCE) of 61.6% which are higher than for the cells with E‐baseline (184.5 mAh g^−1^, 58.8%, respectively) (Figure 3). While there is a slightly higher FCE for the ACSE, the FCEs for both cases are still low. This could be associated with the impurities on the LMTO cathode (discussed later in the text) causing irreversible capacity loss at the first cycle. After the formation cycles, the CE increases reaching an average of 98.8% for ACSE which is higher than 97.9% for E‐baseline at a charge/discharge current density of 20 mA g^−1^ (Figure 3b). Additionally, at the 1^st^ cycle at 20 mA g^−1^ following the four formation cycles at 10 mA g^−1^, the cells with E‐baseline deliver a specific capacity of 164.1 mAh g^−1^ (Figures 3a and Figure S11, Supporting Information). This is then followed by a gradual specific capacity decay with E‐baseline reaching 80% capacity retention after ≈103 cycles (Figure 3a). The cells with ACSE on the other hand deliver a high capacity of 178.9 mAh g^−1^ with improved stability reaching 80% capacity retention after ≈160 cycles. After 200 cycles, the cells with ACSE retain a capacity of 135 mAh g^−1^ and a CE of 99.2%, over 127 mAh g^−1^ and 97.9% for the cells with E‐baseline, respectively (Figure 3). The improved cycling performance of the Li||LMTO cells with ACSE over with E‐baseline is originated from the solvation structure of this ACSE allowing it to exhibit high oxidation potential (Figure S9c and Table S3, Supporting Information) and formation of stable interphases (which will be elaborated later).

### 2.3 Gassing Analysis of Li||LMTO Cells

Another critical issue that is detrimental to the performance of Li||LMTO cells at high charge cutoff voltages is the gas evolution emanating from the electrolyte decomposition and/or the DRX cathode. To analyze gases evolved in cells employing each electrolyte, differential electrochemical mass spectrometry (DEMS) was carried out on Li||LMTO cells with ACSE and E‐baseline and the results are shown in Figure S12 (Supporting Information) and summarized in **Table** **1**. CO_2_ evolution originates from both electrolyte and Li_2_CO_3_ oxidation, where Li_2_CO_3_ exists as an impurity remaining after synthesis of the LMTO; an acid titration^[^ ^15^ ^]^ indicates a fairly high 770 µmol‐Li_2_CO_3_ g^−1^_‐_LMTO or 5.7 wt.% Li_2_CO_3_ in the as‐prepared LMTO electrodes. Partial oxidation of Li_2_CO_3_ primarily occurs over the first 2–3 cycles,^[^ ^16^ ^]^ although, without isotopic labeling, it is difficult to deconvolute the relative CO_2_ evolution contributions between Li_2_CO_3_ oxidation and electrolyte degradation. Furthermore, a complex link exists between electrolyte oxidation and the amount of Li_2_CO_3_ impurity due to reactive oxygen species formation during Li_2_CO_3_ oxidation,^[^ ^17^ ^]^ which further convolutes the exact CO_2_ evolution assignments. Nevertheless, more CO_2_ is evolved in each electrolyte than the amount of Li_2_CO_3_ initially present in the electrode, indicating both electrolytes oxidize at high potentials. ACSE has a slightly lower total CO_2_ evolution over the first 5 cycles, suggesting it may have a slightly enhanced oxidative stability compared to the E‐Baseline, in agreement with the spectroscopic characterization discussed below. Curiously, ACSE exhibits modest H_2_ evolution (Figure S12, Supporting Information) on each of the first 5 cycles, which we ascribe to protic species formation at high potentials followed by diffusion to the Li metal counter electrode to react and evolve H_2_. While the origin of this H_2_ evolution, and whether it occurs due to the same process(es) as the CO_2_ evolution, is currently unknown, further studies on high‐voltage proton abstraction from TTE (the likely origin of the oxidized protic species) are warranted.

**Table 1:** Cumulative CO_2_ outgassing per cycle in µmol g^−1^_‐LMTO_ for Li||LMTO cells using E‐baseline and ACSE.

Electrolyte	Cycle 1	Cycle 2	Cycle 3	Cycle 4	Cycle 5	Cumulative
E‐Baseline	1205.1	220.1	110.5	51.4	31.2	1618.3
ACSE	1161.0	148.8	79.7	63.9	60.6	1514.0

^a)^770 µmol‐Li_2_CO_3_ g^−1^_‐LMTO_ is initially present on the DRX electrode due to synthesis impurities, which contributes significantly to the CO_2_ evolved in the first few cycles.

### 2.4 Postmortem Analyses of Cycled LMTO Cathodes

**Figure 4:**
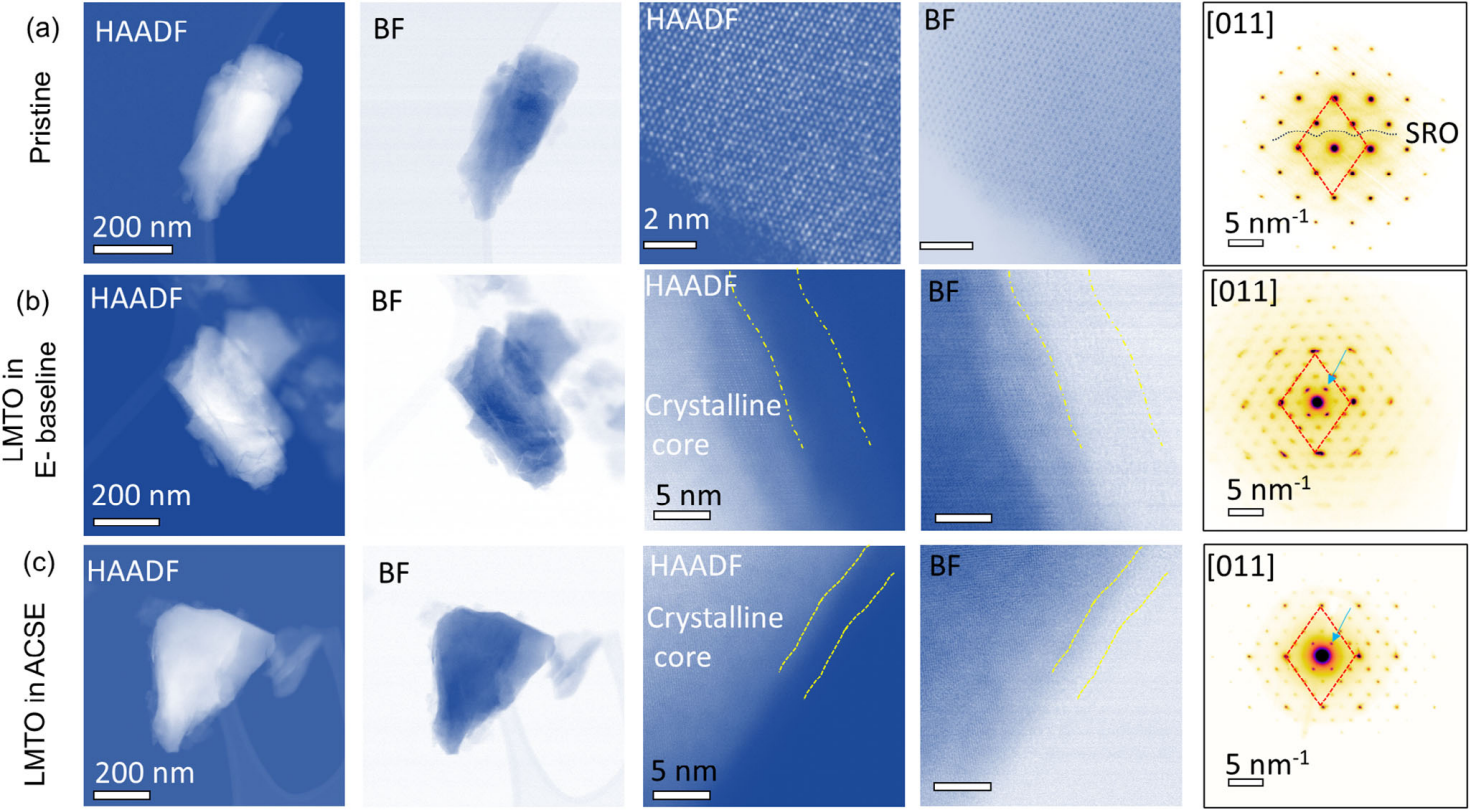
HAADF and BF STEM images and [011] diffraction of a) pristine LMTO particle, b) LMTO particle cycled with E‐baseline, and c) LMTO particle cycled with ACSE (after 200 cycles).

The effects of ACSE on the evolution of the electrode surface and the bulk structure of the LMTO cathode after cycling in comparison to E‐baseline were investigated by conducting scanning transmission electron microscopy (STEM) characterization on the pristine and the cycled LMTO electrodes collected at the 200^th^ cycle discharge state. The high‐angle annular dark‐field (HAADF) and bright‐field (BF) STEM images are shown in **Figure** **4**. Compared to the integral solid bulk structure and the clean surface of the pristine LMTO particle (Figure 4a), the LMTO cycled in E‐baseline exhibits serious cracking in the bulk structure, formation of a ≈ 5 nm cation mixing layer, and ≈6 nm CEI on the surface of the LMTO particle (Figure 4b). However, the LMTO cycled in ACSE maintains the structural integrity after 200 cycles while the cation mixing layer and the CEI are ≈3 nm, respectively (Figure 4c). The STEM results demonstrate that the compatibility and stability between LMTO and ACSE are better than those for LMTO and E‐baseline, which corroborates with the increased specific capacity and longer cycle life for Li||LMTO cells with ACSE than for E‐baseline as shown in Figure 3 and the gassing amount exhibited in Figure S12 (Supporting Information).

**Figure 5:**
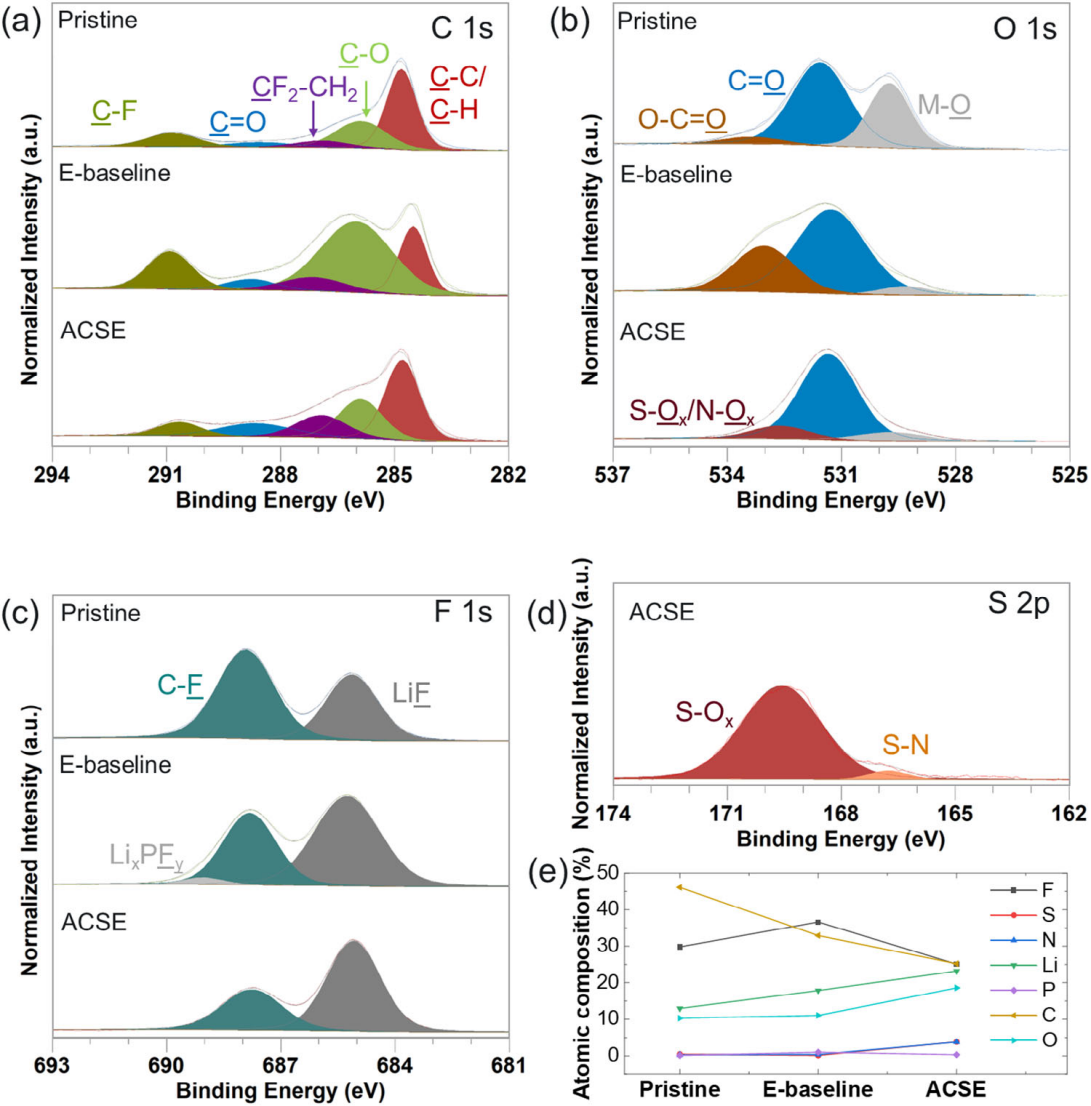
XPS spectra of a) C 1s, b) O 1s, c) F 1s and d) S 2p of pristine LMTO and LMTO cycled with E‐baseline and ACSE. e) The atomic composition of elements on the surfaces of pristine LMTO and cycled LMTO with E‐baseline and ACSE.

On the other hand, the elemental distributions in the pristine LMTO and the cycled LMTO with E‐baseline and ACSE are presented in Figure S13 (Supporting Information). It is seen that manganese (Mn), titanium (Ti), Oxygen (O), and fluorine (F) are uniformly distributed along the surfaces of these LMTO particles. However, the carbon (C) seems not uniform, which may come from the conductive carbon and poly(vinylidene fluoride) (PVDF) binder used in the electrode and the decomposition of the solvents. Therefore, the X‐ray photoelectron spectroscopy (XPS) analysis was conducted to further characterize the surfaces of these LMTO electrodes. **Figure** **5** presents the XPS spectra of C 1s, O 1s, F 1s, and sulfur (S) 2p of the surfaces of the pristine LMTO and the LMTO cycled with E‐baseline and ACSE. The C 1s and O 1s spectra in Figure 5a,b reveal notable peaks including signals for conductive carbon (C─C/C─H, 284.8 eV, C 1s), PVDF (C─F, ≈ 290.7 eV and CF_2_‐CH_2_, ≈ 287.1 eV, C 1s), and (C═O, 289.0 eV, C 1s and 531.1 eV, O 1s) on the pristine LMTO surface. Additionally, the F 1s spectrum of the pristine LMTO shows a prominent C─F peak (≈687.9 eV, F 1s) from PVDF binder and a notable LiF peak (≈685.4 eV, F 1s), the latter being attributed to the residual LiF impurity in the as‐prepared LMTO electrode. This is because similar to the LMTO used in our previous work,^[^ ^10^ ^]^ the present LMTO was also calcined with LiF with the desire to achieve F‐doping to enhance the stability of the LMTO as reported previously.^[^ ^18^ ^]^ However, the F was not incorporated into the bulk structure instead remained as a residual LiF impurity at the LMTO surface (Figure S2, Supporting Information).

For the cycled LMTO electrodes, the C 1s spectra reveal similar compositions on LMTO cycled with E‐baseline and ACSE. However, the C─O peak is magnified in the E‐baseline cycled surface suggesting the formation of less stable CEI rich in organic species in E‐baseline. Additionally, analysis of the O 1 s and S 2p spectra in Figure 5b,d reveals the presence of S‐O_x_/N‐O_x_ (≈532.2 eV, O 1s) and S‐N (≈170 eV, S 2p) peaks on the ACSE‐derived CEI, indicating the decomposition of the LiFSI salt. These are known to enhance the mechanical robustness and uniformity of the CEI layer contributing to improved cycling stability.^[^ ^13^, ^19^ ^]^ In contrast the CEI formed in E‐baseline exhibits LiPF_6_ derived species, as indicated by the Li_x_PF_y_ (685.6 eV, F 1s) peak in Figure 5c. The decomposition of the LiPF_6_ salt anions is associated with corrosion effects due to the trace amount of HF in the E‐baseline which compromises the structural integrity and overall stability of the LMTO cathode, aligning with the earlier observation from the STEM analysis. Furthermore, the atomic composition data (Figure 5e) reveal that the CEI formed in ACSE cycled LMTO contains notably lower levels of C and F species compared with that in E‐baseline, suggesting reduced decomposition of electrolyte components in ACSE cells, and highlighting the superior stability and quality of the CEI generated in ACSE.

The XPS analysis of the Li metal anode surfaces was also conducted, and the results are presented in Figure S14 (Supporting Information). It is observed that the solid electrolyte interphase (SEI) layer formed in E‐baseline is dominated by a LiPF_6_ derived species Li_x_PO_y_F_z_ (≈685.5 eV) reflecting the decomposition of LiPF_6_ salt. In contrast, the SEI formed in ACSE shows predominant signals for diluent‐derived C─F (687.9 eV, F 1s) and LiF (≈685 eV, F 1s) (Figure S14, Supporting Information). These SEI constituents from ACSE efficiently suppress further solvent decomposition during charge/discharge cycles, thereby enhancing the stability of the Li metal anode. These findings reveal that ACSE not only effectively passivates the LMTO cathode, reducing structural degradation, but also forms a stable and robust SEI on the Li metal anode, thus leading to enhanced battery cycling stability over E‐baseline.

## 3 Conclusion

In this study, we employed a combination of computational simulations and experimental methods to systematically investigate the solvation structures and properties of different electrolytes with varying solvent‐to‐diluent ratios. This approach provides a foundation for designing advanced controlled‐solvation electrolytes tailored to enhance the performance of high‐voltage high‐capacity DRX cathodes. The results show that the ACSE (LiFSI‐1.6DMC‐3TTE, by mol) exhibits significantly reduced free solvent molecules resulting in the formation of a solvation structure with large aggregates. Due to this unique solvation structure, the ACSE shows high oxidation stability (≈5.1 V vs Li/Li^+^) and high cathodic stability (99.6% average Li CE), thus enables the formation of stable and robust CEI and SEI enriched in salt anion derived species on the cathode and anode surfaces. Such a CEI effectively reduces the continuous electrolyte decomposition reactions and protects the LMTO cathode particles against bulk structural degradation and cracking, and consequently, Li||LMTO cells with ACSE demonstrate enhanced cycling performance. Specifically, the Li||LMTO cells with the ACSE deliver a higher initial specific capacity of 202.6 mAhg^−1^ compared with 184.5 mAhg^−1^ for the cells with the state‐of‐the‐art electrolyte (E‐baseline) at a charge/discharge rate of 10 mA g^−1^. Furthermore, the cells with ACSE exhibit a cycle life almost double that of the E‐baseline cells. These findings suggest that by carefully engineering the electrolyte structure with the aid of simulations and calculations we can mitigate common issues associated with DRX cathodes, thereby facilitating the practical use of DRX cathodes like LMTO in high‐energy density batteries. This work underscores the potential of advanced electrolyte design as a critical factor for the commercialization of DRX cathodes in next‐generation energy storage systems.

## 4 Experimental Section

### Electrolyte and Electrode Preparation

The different electrolytes studied in this work were prepared by dissolving the salt LiFSI in a mixture of DMC and TTE in the respective mol fractions shown in Table S2 (Supporting Information). The baseline electrolyte (E‐baseline) of 1 M LiPF_6_ in EC‐DMC (1:2 by wt.) was formed by dissolving LiPF_6_ salt in a mixture of EC and DMC. All the electrolyte preparation was done in an argon (Ar)‐filled MBraun glovebox (O < 1 ppm, H_2_O < 1 ppm). The Li_1.094_Mn_0.676_Ti_0.228_O_2_ (LMTO) powder was synthesized according to the procedure under the supplementary experimental section. The LMTO electrodes were fabricated using a composition of 75 wt.% carbon‐coated LMTO powder as the active material, 10 wt.% Denka black, 5 wt.% carbon nanotube and 10 wt.% PVDF. The LMTO electrode laminates (mass loading = 4.8 mg cm^−2^) were punched into disks (diameter = 1.27 cm) and dried in a vacuum at a temperature of 130 °C overnight before use. Li metal chips (thickness = 250 µm and diameter = 1.55 cm) purchased from MTI corporation USA were used as received.

### Electrochemical Tests

The ionic conductivity measurements were conducted using custom‐built coin cells and the electrochemical impedance spectroscopy (EIS) spectra were taken in the frequency range of 100 mHz to 100 kHz on Bio‐Logic SAS (VMP‐300) at 25 °C. The dissociation degree for each electrolyte was obtained as described in prior work.^[^ ^20^ ^]^ The oxidation potentials of the different electrolytes were measured by linear sweep voltammetry (LSV) on Bio‐Logic SAS (VMP‐300) using Li as the reference and counter electrode and Super P coated on Al foil as the working electrode.

The Li||LMTO coin cells were assembled using a piece of LMTO cathode, a 250‐µm thick Li metal anode, a polyethylene (PE) separator, and 75 µL of electrolyte for each cell. During cell assembly, a positive case coated with Al with an additional piece of Al foil (1.90 cm diameter) placed between the positive case and the LMTO cathode were used to prevent corrosion of the stainless steel by the electrolytes. The cells were cycled on Landt battery testers at a charge/discharge current density of 10 mA g^−1^ for the first four formation cycles, followed by cycling at a charge/discharge current density of 20 mA g^−1^. The coin cells were placed in Neware temperature chambers of 30 °C and the test was conducted in the voltage range between 2.0 and 4.8 V.

Differential Electrochemical Mass Spectrometry (DEMS) was used to measure outgassing as described in previous studies.^[^ ^21^ ^]^ Custom‐built Swagelok cells with inlet and outlet capillaries were used for gas flow. In an Ar‐filled glovebox, the cells were assembled with a Li foil anode (FMC), a quartz microfiber separator (Whatman), and an LMTO cathode. For each DEMS experiment, 60 µL of electrolyte was used. Above the cathode was an Al mesh disc and two Al rings to create a headspace (≈100 µL) for gas accumulation. Once assembled, the Swagelok cells were attached to the DEMS system and the pressure was monitored to ensure no leaks. Every 10 min, a small (500 µL) pulse of Ar gas was used to purge the cell headspace and then sent to the mass spectrometer (MS) gas analyzer. MS signals were calibrated for CO_2_, in Ar carrier gas to allow for gas quantification. Cells were cycled between 4.8 and 2.0 V at a current of 0.1 mol Li h^−1^ using a Bio‐Logic VSP‐series potentiostat.

### Pulsed‐Field Gradient Nuclear Magnetic Resonance (PFG‐NMR) Spectroscopy

The diffusivity values and the Li^+^ transference number for each electrolyte were measured using PFG‐NMR technique. ^1^H, ^7^Li, and ^19^F diffusion measurements were performed on a Bruker Avance NEO spectrometer operating at 500 MHz (11.7 T). The 90° pulse widths were 8.4 µs for ^1^H, 15.3 µs for ^7^Li, and 18.5 µs for ^19^F. To reduce the convection effect and maintain a stable lock signal during measurements, samples were loaded into 3 mm NMR tubes, which were then inserted inside 5 mm NMR tube containing DMSO‐d_6_ for locking and shimming. Diffusion measurements employed a bipolar gradient pulse with stimulated echo and LED (“ledbpgp2s”) pulse sequence, using diffusion times of 200, 500, and 400 ms, and gradient pulse widths of 2, 4, and 3 ms for ^1^H, ^7^Li, and ^19^F, respectively. Experiments were conducted with 16 gradient strength increments, 8 scans per increment, and 32 steady‐state scans. The gradient field strength ranged from 1 to 45 G cm^−1^, ensuring that the signal of the final increment was reduced to less than 10% at the highest gradient.

### Solid‐State Nuclear Magnetic Resonance (NMR) Spectroscopy

Solid‐state NMR spectra of the pristine LMTO powder were acquired at a low magnetic field of 2.35 T (100 MHz for ^1^H) using a wide‐bore Bruker BioSpin spectrometer equipped with a DMX 500 MHz console and a custom‐made 1.3 mm, single channel broadband magic angle spinning (MAS) probe tuned to either ^7^Li (38.9 MHz) or ^19^F (94.1 MHz). Spectra were obtained using a rotor‐synchronized spin‐echo sequence (90° − *t*_R_ − 180° − *t*_R_) with 90° radio frequency (RF) pulses of 0.45 µs for ^7^Li and of 0.3 µs for ^19^F. ^7^Li chemical shifts were externally referenced against a 1 m aqueous LiCl solution (δ_iso_ = 0 ppm). ^19^F chemical shifts were referenced against a 1 m aqueous NaF (^19^F δ_iso_ = −118.14 ppm) solution. A recycle delay of 20 s was used throughout, sufficiently long for all ^7^Li and ^19^F spins within the sample to re‐equilibrate between scans. The sample was packed in a 1.3 mm rotor in an Ar‐filled glovebox, and the rotor was spun at 60 kHz MAS during data acquisition using dry nitrogen. The NMR data was processed using the Bruker TopSpin 3.6.0 software.

### Scanning Transmission Electron Microscopy (STEM)

The STEM images were obtained by dispersing both pristine and cycled particles onto TEM lacey carbon grids in an Ar‐filled glovebox. A Titan 80–300TM scanning/transmission electron microscope operated at 300 kV was used to obtain the electron diffraction images. Also, a Spectra Ultra S/TEM with a new generation Ultra‐X EDS detector at an acceleration voltage of 300 kV was used to collect HAADF/BF imaging and EDS data. The imaging and EDS data were acquired with the Ultra‐X instrument using a probe current of 30 pA to prevent major beam damage.

### X‐Ray Photoelectron Spectroscopy (XPS)

A Physical Electronics Quantera Scanning X‐ray Microprobe (Physical Electronics, Germany), with a focused monochromatic Al Kα (1486.7 eV) source for excitation was used for the XPS analysis. The survey scans were collected, with a pass‐energy of 140 eV and a step size of 0.5 eV in order to quantify the atomic composition of elements on the surface. A pass‐energy of 69 eV with a step size of 0.125 eV was used to collect high‐energy resolution (C1s, O1s, F1s, S2p, and P2p) spectra. The fitting of the collected spectra was done on CasaXPS software calibrated with C 1s at 284.8 eV.

### Viscosity Measurement

The measurement of the viscosity of the electrolytes as a function of temperature was conducted using an Anton Paar rheometer (MCR‐101; Ashland, VA, USA), and the utilization of a double gap measuring system DG26.7 SS coupled with a C‐PTD200 cell. A Peltier system integrated into the rheometer was used for temperature control and a temperature ramp was applied to the electrolyte samples during measurements. The temperature was set at 0 °C to start the measurements and increased linearly with time from 0 to 60 °C for a total time of 45 min while the viscosity was measured and recorded with the values logged in every 30 sec. A constant shear rate of 50 1/s was used for all measurements. The DG26.7 measuring system was dissembled for washing and drying after each sample measurement and then reassembled for a new sample. A nitrogen gas was passed through the measuring instrument to reduce the exposure of samples to the air.

### Molecular Dynamics Simulations

The solvation structures and the transport properties of the designed electrolytes were calculated using MD trajectories. All classical MD simulations were performed in the LAMMPS code.^[^ ^22^ ^]^ The Optimized Potentials for Liquid Simulations All‐Atom (OPLS‐AA) force field^[^ ^23^ ^]^ was employed with fitted parameters for the salt LiFSI,^[^ ^24^ ^]^ the solvent DMC,^[^ ^12a^ ^]^ and the diluent TTE,^[^ ^25^ ^]^ respectively. The anions, cations, diluents, and solvent particles were randomly packed into a cubic simulation box using Packmol.^[^ ^26^ ^]^ The atomic charges for all species in this work were calculated using the Density Functional Theory (DFT) package Orca^[^ ^27^ ^]^ with the 6–31++G(d,p) basis set and long‐range corrected Becke–Lee–Yang–Parr functional followed by RESP analysis implemented in the Multiwfn^[^ ^28^ ^]^ package. Partial charges of the ionic species were scaled by a factor of 0.72 to account for the fact that ion−ion interactions were typically overestimated in nonpolarizable force fields.^[^ ^29^ ^]^ Each simulation consisted of ≈45000 atoms, with the exact number varied slightly to precisely reach the target concentration. In each simulation, the equations of motion were numerically integrated using the velocity‐Verlet algorithm. Each system was periodic in the *x*, *y*, and *z* directions and incorporated the PPPM^[^ ^30^ ^]^ method with an accuracy of 1.0×10^−4^ to compute long‐range Coulombic interactions. The as‐prepared system was equilibrated using a conjugate gradient energy minimization. The system was then equilibrated in the canonical (NVT) ensemble at a pressure of 1 atm and temperature of 400 K for 1 ns, followed isothermal−isobaric (NPT) ensemble at a pressure of 1 atm and temperature of 400 K for 1 ns. Then the temperature of the system was gradually reduced to 298 K at a pressure of 1 atm for 7 ns. Production runs were subsequently carried out in the canonical (NVT) ensemble at 298 K using the Nosé−Hoover‐style thermostat for 40 ns with a timestep of 1 fs. Simulations were carried out for 49 ns with the last 30 ns used for analysis with MD Analysis^[^ ^31^ ^]^ and Solvation Analysis.^[^ ^12b^ ^]^

The transport properties were computed based on the Onsager transport coefficients, *L_ij_* and *L_ii_* ^self^ calculated from the MD simulations. The *L_ij_* term captures the total flux correlations between species *i* and *j* in the system. The *L_ii_* ^self^ term corresponds to the ideal contributions to the transport and was related to the self‐diffusion coefficient (*D_i_*) as *L_ii_* ^self^ = *D_i_c_i_*/*k_B_T* where *c_i_* is the concentration of the species i in the system, and *k_B_* is the Boltzmann constant. The *L_ij_* terms can be computed based on the Green‐Kubo relations which are elaborately discussed in Fong et al.^[^ ^32^ ^]^ With the *L_ij_* terms computed, the ionic conductivity and the ideal transference number of Li^+^ ions of the system were computed as follows:

(1) $$\sigma =F^{2}\;\left(L_{++}+L_{--}-2L_{+-}\right)$$

(2) $$t_{Li}^{ideal}=\frac{L_{++}^{self}}{L_{++}^{self}+L_{--}^{self}}$$

where *L*_++_ represents the Onsager transport coefficient for Li^+^ – Li^+^ ion correlation, *L*_−_ represents the Onsager transport coefficient for FSI^− –^ FSI^−^ ion correlation and *L*_+−_ represents the Onsager transport coefficient for Li^+^ – FSI^−^ ion correlations.

### Density Functional Theory Calculations

The oxidation potentials of DMC, FSI^−^, and the Li^+^‐*a*DMC‐*b*FSI^−^ complexes were calculated with the Q‐Chem package. All the DFT calculations in this work were carried out with ωB97X‐D3 functional^[^ ^33^ ^]^ and the def2‐SVPD basis set.^[^ ^34^ ^]^ The solvation effect was implicitly accounted for with the Polarizable Continuum Model (PCM),^[^ ^35^ ^]^ where the dielectric constant was set to be 4.65, an average of that of DMC (3.107) and TTE (6.20), to approximate the solvation environments considered in this work. All the molecules were subject to geometry optimization, with the energy and force convergence thresholds being 1×10^−6^ Hartree and 3×10^−4^ Hartree/Bohr, respectively. For the complexes, the oxidation potentials in the Li^+^/Li scale were calculated as (*E*_+_ – *E*_0_)/*F* – 1.4 V, where *E*_+_ and *E*_0_ respectively refer to the energies of the oxidized and unoxidized molecule at 298.15K, and *F* is Faraday's constant. For DMC and FSI^−^, the energies were replaced by the free energies.

## Conflict of Interest

The authors declare no conflict of interest.

## Supporting information



Supporting Information

## Data Availability

The data that support the findings of this study are available from the corresponding author upon reasonable request.
