# Supplementary material for: Designing Advanced Electrolytes for High‐Voltage High‐Capacity Disordered Rocksalt Cathodes
Source: Small. 2025 Mar 24;21(18):2501600. doi: 10.1002/smll.202501600 (PMC12051754; doi:10.1002/smll.202501600)
Supplement: Supplementary file 1 — Supporting Information [file SMLL-21-2501600-s001.docx]

Supporting Information

Designing Advanced Electrolytes for High-Voltage High-Capacity Disordered Rocksalt Cathodes

Ridwan A. Ahmed, Rohith Srinivaas Mohanakrishnan, Jingyang Wang, Krishna P. Koirala, Qian Zhao, Yanbao Fu, Ying Chen, Justin C. Rastinejad, Tianyu Li, Lirong Zhong, Mateusz Zuba, Carrie Siu, Ozgenur Kahvecioglu, Raphaële J. Clément, Bryan D. McCloskey, Vincent S. Battaglia, Kristin Persson*, Chongmin Wang* and Wu Xu*

* Emails: [kapersson@lbl.gov](mailto:kapersson@lbl.gov); [chongmin.wang@pnnl.gov](mailto:chongmin.wang@pnnl.gov); [wu.xu@pnnl.gov](mailto:wu.xu@pnnl.gov)

**Supplementary Experimental Section**

*Baseline LMTO Synthesis*

The synthesis of the targeted disordered rock salt (DRX) compound Li₁.₂Mn₀.₆Ti₀.₂O₁.₈F₀.₂ was achieved using Mn₂O₃, TiO₂, Li₂CO₃, and LiF as precursors. The precursor compositions were Mn₂O₃ at 30 mol%, TiO₂ at 20 mol%, Li₂CO₃ at 59 mol% (including a 9 mol% excess to offset Li-loss during high-temperature calcination), and LiF at 20 mol%. A total mass of approximately 200 g of these precursors was combined and ball-milled with yttria-stabilized zirconia (Y-stabilized ZrO₂) in 500 mL jars at 100 RPM using a Retsch Planetary Ball Mill PM 400.

The ball-milled mixture was then calcined at 1000 °C for 4 hours with a ramp rate of 5 °C/min under an argon (Ar) atmosphere. The calcination was performed as a loose powder at a scale of 30 g. Following this, the calcined DRX material was ball-milled again at 400 RPM in an organic solvent for 4 hours without any carbon. The processed material was then recovered, dried, and sieved using a 45-micron mesh.

X-ray diffraction (XRD) and ^19^F solid-state nuclear magnetic resonance (NMR) analyses revealed the presence of LiF impurities, with minimal to no fluorine content in the bulk structure, as shown in Figures S1 and S2. The Li, Mn, Ti, and F contents in the synthesized DRX powder were determined using inductively coupled plasma - optical emission spectrometry (ICP-OES) and fluoride ion-selective electrode (F-ISE), with the results presented in Table S1. The ^7^Li solid-state NMR data (Figure S2b) indicated that the actual lithium content in the bulk DRX is 0.872.

Therefore, the final composition of the synthesized powder was determined to be The Li_1.094_Mn_0.676_Ti_0.228_O_2_, with no fluorine in the bulk structure. This compound is abbreviated as LMTO. The synthesis and most of the analytical work on the pristine DRX material were conducted at the Argonne National Laboratory (ANL) facility.

**Figure S1.** X-ray diffraction of the pristine DRX powder. The presence of LiF impurity is evident in the synthesized DRX powder.


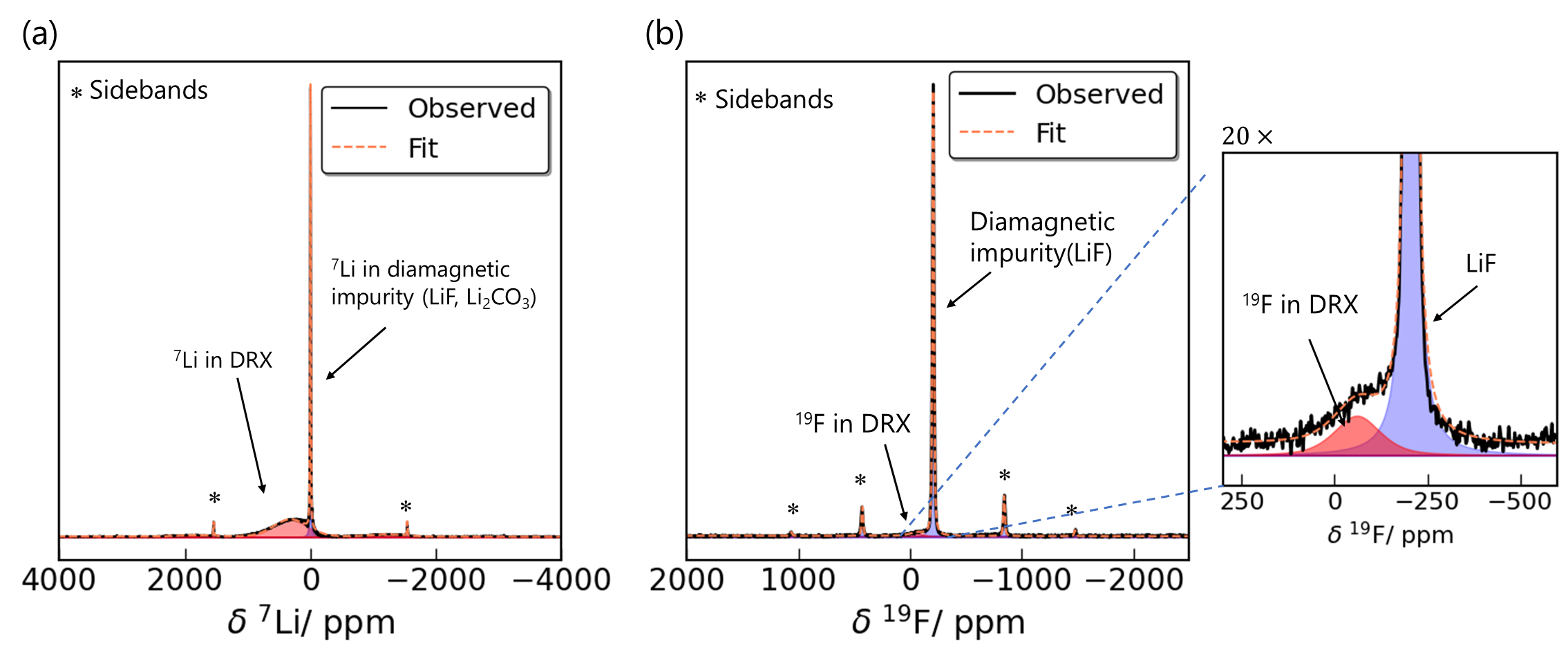


**Figure S2.** (a) ^7^Li solid-state NMR spectra and fit of pristine DRX powder. (b) ^19^F solid-state NMR spectra and fit of pristine DRX powder.

Two major components are noted in Figure S2a: Sharp resonance at 0 ppm corresponds to ^7^Li in the diamagnetic impurities (LiF and Li_2_CO_3_) while the broad band corresponds to ^7^Li in the DRX. Fit indicates that ~32% Li impurity. In Figure S2b, two major components are noted: Sharp resonance at -204 ppm corresponds to ^19^F in LiF. Broad band corresponds to ^19^F in DRX. The signal attributed to F in DRX is extremely minor compared to LiF impurity, suggesting most of F in the sample exist as F impurity. Less than 5% integrated intensity corresponds to F in DRX according to fit. The relaxation delay for each solid-state NMR spectrum was 20 s.

**Table S1.** Li, Mn, Ti, and F stoichiometries determined by ICP-OES, F-ISE and solid-state NMR, using a method detailed in our previous work.^[1]^

| Element | Li | Mn | Ti | F |
| --- | --- | --- | --- | --- |
| ICP-OES/F-ISE | 1.283 | 0.537 | 0.180 | - |
| ssNMR | 0.872* |  |  | ~0.05 |

The composition obtained from ICP and F-ISE results corresponds to the entire composition of the sample including the impurity phases. This actual Li content in the bulk DRX (0.872) was obtained by mutiplying the total Li content in the sample by the fraction of ^7^Li solid-state NMR signal attributed to Li in the bulk DRX phase (paramagnetically broadened resonance, as described in prior work).^[1]^ Since ^19^F ssNMR indicates a negligible amount of F incorporated into the bulk DRX structure, F was omitted in the final DRX composition.

**Figure S3.** Frequency ratios of various solvation species present in the electrolytes of LiFSI-1.6DMC-*y*TTE (by mol) where (a) *y* = 1, (b) *y* = 2, and (c) *y* = 3.

At 1.6DMC, the majority of the solvation species are 2FSI-2DMC, followed by 3FSI-1DMC, then by 1FSI-3DMC. Increasing the TTE content has an almost negligible effect on the populations of the solvation shell compositions.

**Figure S4.** Frequency ratios of various solvation species present in the electrolytes of LiFSI-2DMC-*y*TTE (by mol) where (a) *y* = 1, (b) *y* = 2, and (c) *y* = 3.

At 2DMC, the majority of the solvation species is 2FSI-2DMC, followed by 1FSI-3DMC, then by 3FSI-1DMC. Increasing the TTE content has an almost negligible effect on the populations of the solvation shell compositions.

**Figure S5.** Frequency ratios of various solvation species present in the electrolytes of LiFSI-3DMC-*y*TTE (by mol) where (a) *y* = 1, (b) *y* = 2, and (c) *y* = 3.

At 3DMC, the majority of the solvation species are 1FSI-3DMC, followed by 2FSI-2DMC, then by 0FSI-4DMC. Increasing the TTE content in the solvent mixture slightly decreases the amount of 0FSI-4DMC relative to that of 2FSI-2DMC.

**Figure S6.** Frequency ratios of various solvation species present in the electrolytes of LiFSI-4DMC-*y*TTE (by mol) where (a) *y* = 1, (b) *y* = 2, and (c) *y* = 3.

At 4DMC, the majority of the solvation species are 1FSI-3DMC, followed by 0FSI-4DMC, then by 2FSI-2DMC. Increasing the TTE content in the solvent mixture increases the amount of 1FSI-3DMC, while decreasing the amount of 0FSI-4DMC.

**Figure S7.** Frequency ratios of various solvation species present in the electrolytes of LiFSI-5DMC-*y*TTE (by mol) where (a) *y* = 1, (b) *y* = 2, and (c) *y* = 3.

At 5DMC, the majority of the solvation species are 0FSI-4DMC, followed by 1FSI-3DMC, then by 2FSI-2DMC. Increasing the TTE content in the solvent mixture increases the amount of 1FSI-3DMC, while decreasing the amount of 0FSI-4DMC.

**
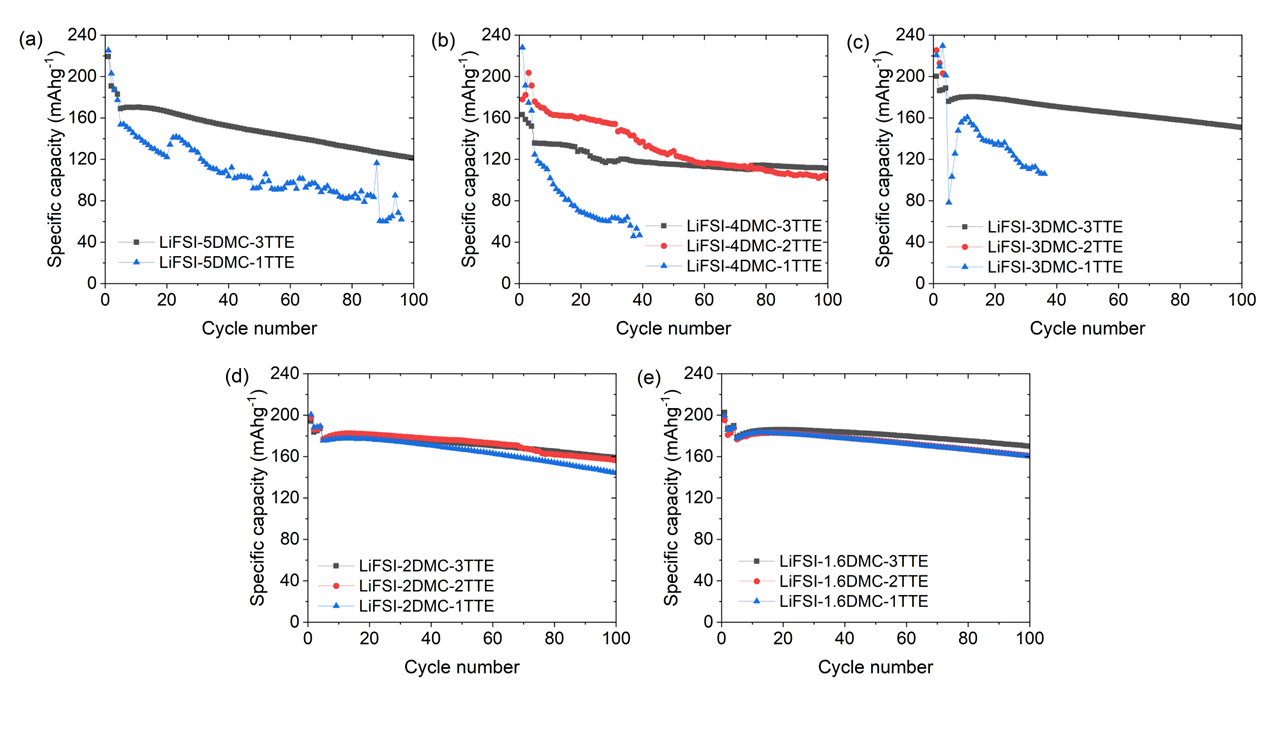
**

**Figure S8.** Cycling performance of the 15 studied electrolytes with the formula LiFSI-xDMC-yTTE (by mol), where x = 5, 4, 3, 2 and 1.6, y = 1, 2 and 3, in Li||LMTO coin cells in the voltage range of 2.0-4.8 V at 20 mA g^-1^ after four formation cycles at 10 mA g^-1^, under 30 °C. Note: The cycling of LiFSI-5DMC-2TTE (by mol) in Figure S8a cannot be obtained, so the result is not included. The reason is unclear yet.


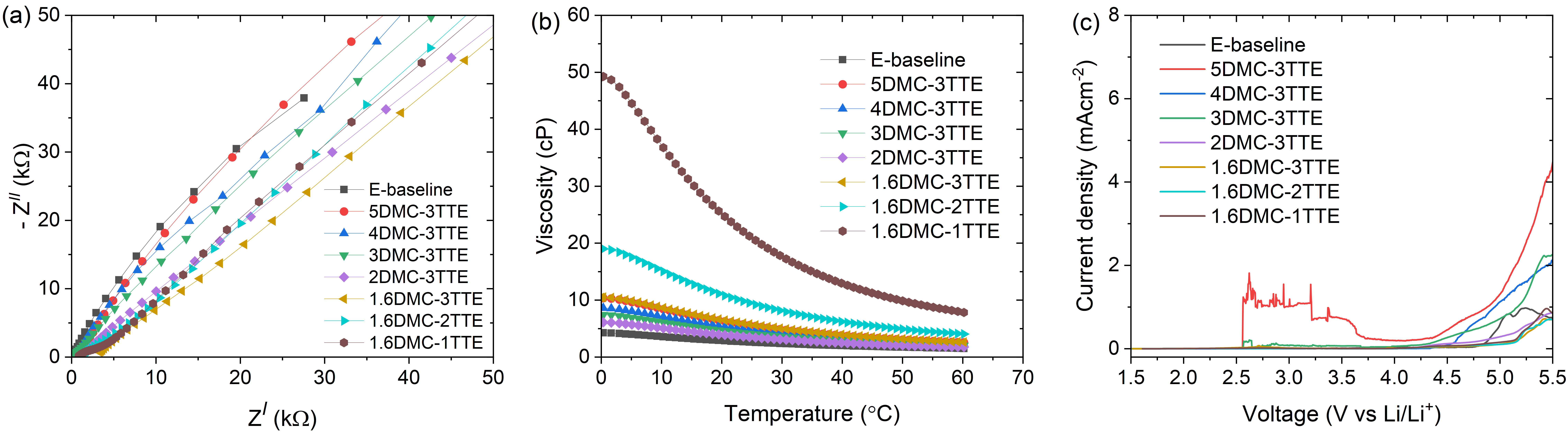


**Figure S9.** Experimental measurements of selected electrolytes and E-baseline (1 M LiPF_6_ in EC-DMC at 1:2 by wt.). (a) EIS spectra for the ionic conductivity measurements at 25 °C. (b) Viscosity versus temperature, (c) LSV curves.

**Table S2.** Calculated transport properties of selected electrolytes from MD simulations

|  | Ionic conductivity  (mS cm^-1^) | D_+_  (× 10^-10^ m^2^ s^-1^) | D_-_  (× 10^-10^ m^2^ s^-1^) | D_DMC_  (× 10^-10^ m^2^ s^-1^) | D_TTE_  (× 10^-10^ m^2^ s^-1^) | Li^+^ transference number |
| --- | --- | --- | --- | --- | --- | --- |
| LiFSI-5DMC-3TTE (by mol) | 5.31 | 1.20 | 1.67 | 2.09 | 3.41 | 0.42 |
| LiFSI-4DMC-3TTE (by mol) | 5.03 | 1.15 | 1.59 | 1.70 | 3.34 | 0.42 |
| LiFSI-3DMC-3TTE (by mol) | 2.94 | 1.07 | 1.36 | 1.38 | 3.42 | 0.44 |
| LiFSI-2DMC-3TTE (by mol) | 2.17 | 0.724 | 0.808 | 0.913 | 4.36 | 0.47 |
| LiFSI-1.6DMC-3TTE (by mol) | 0.78 | 0.515 | 0.534 | 0.726 | 5.11 | 0.49 |
| LiFSI-1.6DMC-2TTE (by mol) | 1.04 | 0.273 | 0.285 | 0.413 | 3.51 | 0.49 |
| LiFSI-1.6DMC-1TTE (by mol) | 1.2 | 0.167 | 0.189 | 0.225 | 1.50 | 0.47 |

Notes: D_+_ represents the diffusion coefficient of Li^+^, D_-_ is the diffusion coefficient of FSI^-^, D_DMC_ is the diffusion coefficient of solvent DMC, D_TTE_ is the diffusion coefficient of diluent TTE.

| Electrolytes | Ionic conductivity  (mS cm^-1^) | Viscosity (cP) | Oxidative potential  (V) | *D*_+_  (× 10^-10^ m^2^ s^-1^) | *D*_-_  (× 10^-10^ m^2^ s^-1^) | *D*_DMC_  (× 10^-10^ m^2^ s^-1^) | *D*_TTE_  (× 10^-10^ m^2^ s^-1^) | Li^+^ transference number | Dissociation degree  (%) |
| --- | --- | --- | --- | --- | --- | --- | --- | --- | --- |
| LiFSI-5DMC-3TTE (by mol) | 4.16 ± 0.16 | 3.42 | ~4.3 | 1.85 | 2.04 | 4.00 | 3.72 | 0.48 | 26.1 |
| LiFSI-4DMC-3TTE (by mol) | 3.59 ± 0.07 | 4.03 | ~4.3 | 1.57 | 1.70 | 3.05 | 3.30 | 0.48 | 22.7 |
| LiFSI-3DMC-3TTE (by mol) | 3.08 ± 0.15 | 4.66 | ~4.3 | 1.39 | 1.46 | 2.50 | 3.08 | 0.49 | 20.6 |
| LiFSI-2DMC-3TTE (by mol) | 2.11 ± 0.05 | 5.44 | ~4.5 | 1.02 | 1.02 | 1.53 | 2.94 | 0.50 | 17.4 |
| LiFSI-1.6DMC-3TTE (by mol) | 0.58± 0.02 | 5.61 | ~5.1 | 0.84 | 0.84 | 1.25 | 3.02 | 0.50 | 5.4 |
| LiFSI-1.6DMC-2TTE (by mol) | 1.17± 0.04 | 9.21 | ~ 5.1 | 0.61 | 0.60 | 0.88 | 2.19 | 0.50 | 11.3 |
| LiFSI-1.6DMC-1TTE (by mol) | 1.46 ± 0.20 | 20.50 | ~5.1 | 0.39 | 0.39 | 0.55 | 1.21 | 0.50 | 14.4 |
| 1 M LiPF_6_ in EC-DMC (1:2 by wt.%) | 10.3 ± 0.01 | 2.55 | ~ 4.8 | 2.31 | 3.26 | 6.29 | 4.32 | 0.42 | 49.6 |

**Table S3.** Basic properties of different electrolytes from experimental measurements

Notes: *D*_+_ represents the diffusion coefficient of Li^+^, *D*_-_ is the diffusion coefficient of FSI^-^, *D*_DMC_ is the diffusion coefficient of solvent DMC, *D*_TTE_ is the diffusion coefficient of diluent TTE.


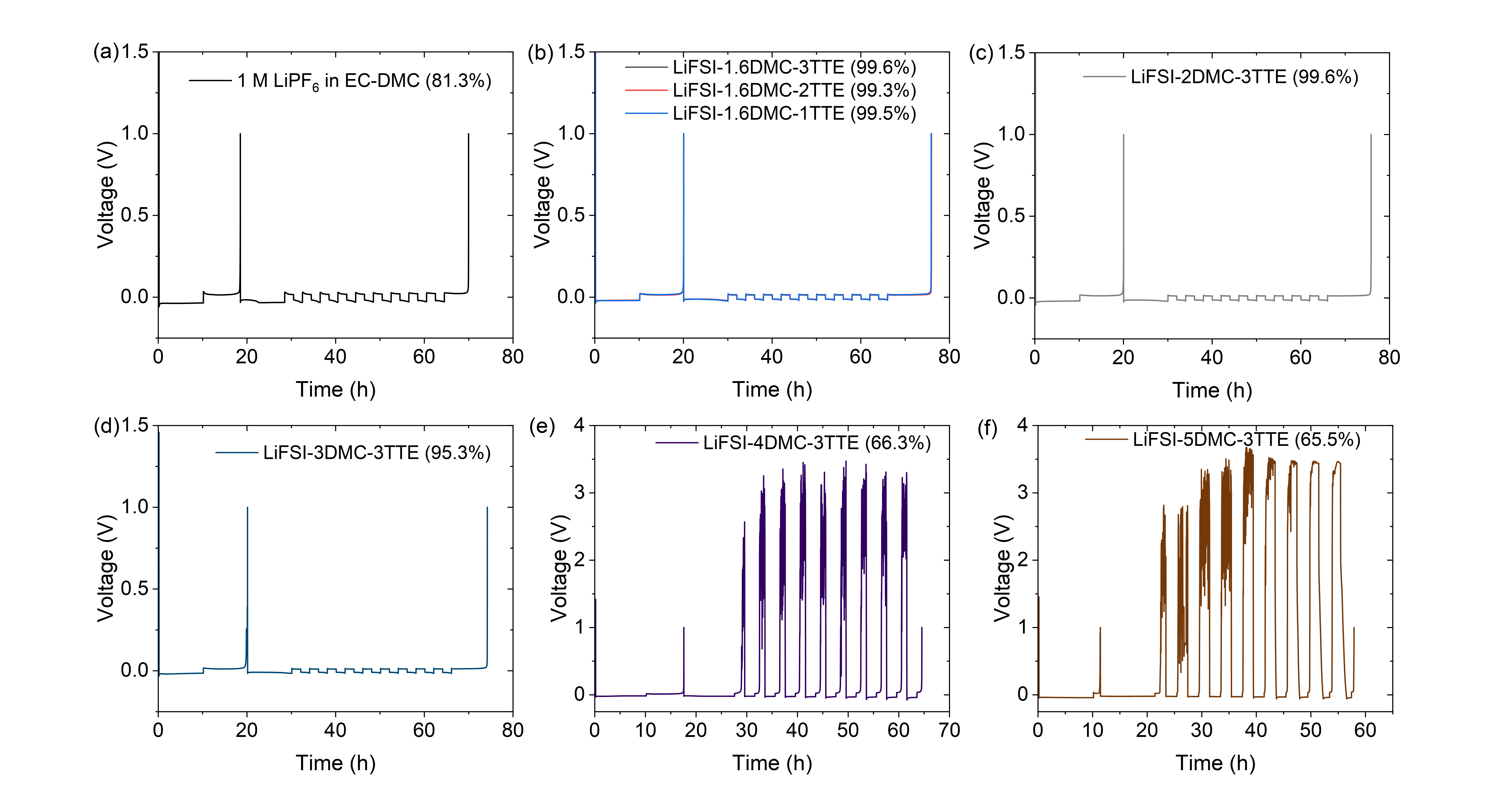


**Figure S10.** Voltage profiles of Li deposition and stripping during average Li CE measurement using the electrolytes in Li||Cu cells. (a) 1 M LiPF_6_ in EC-DMC (E-baseline), (b) LiFSI-1.6DMC-yTTE (where y = 1, 2, 3), (c) LiFSI-2DMC-3TTE, (d) LiFSI-3DMC-3TTE, (e) LiFSI-4DMC-3TTE, and (f) LiFSI-5DMC-3TTE. The values in the parentheses are the average Li CEs of the electrolytes.

*
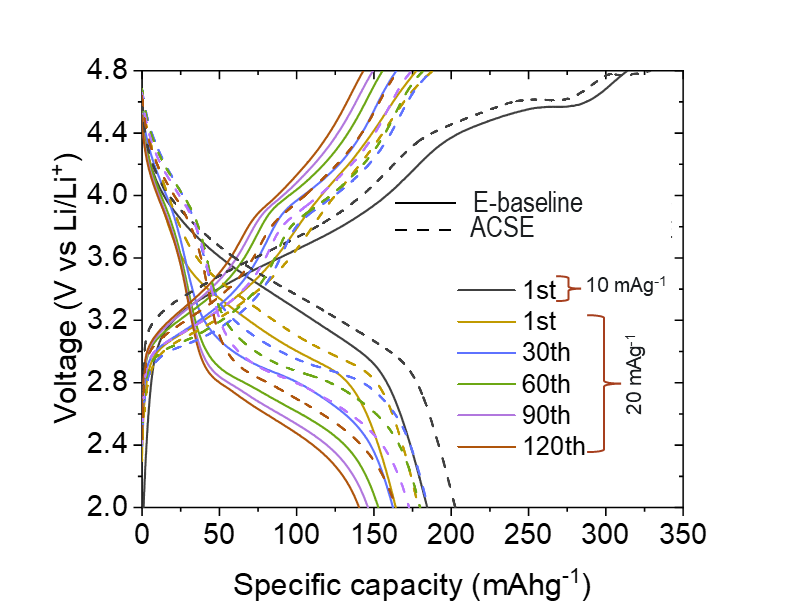
*

**Figure S11.** Charge/discharge voltage profiles at selected cycle numbers for Li||LMTO cells using the ASCE and E-baseline in the voltage range of 2.0-4.8 V.


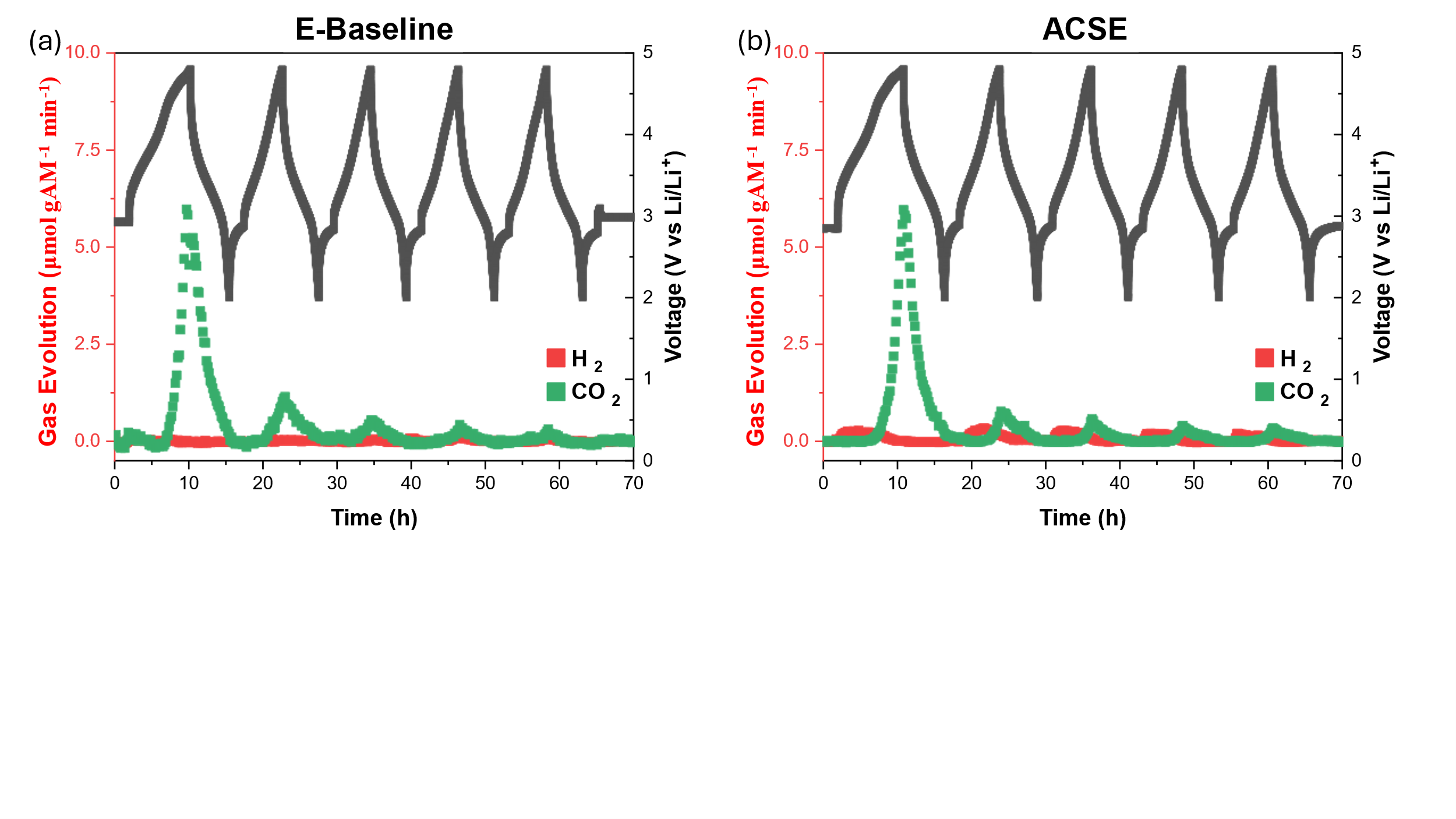


**Figure S12.** DEMS results of Li||LMTO cells with (a) E-Baseline and (b) ACSE cycled between 4.8 V and 2.0 V at 0.1 mol Li^+^ h^-1^.


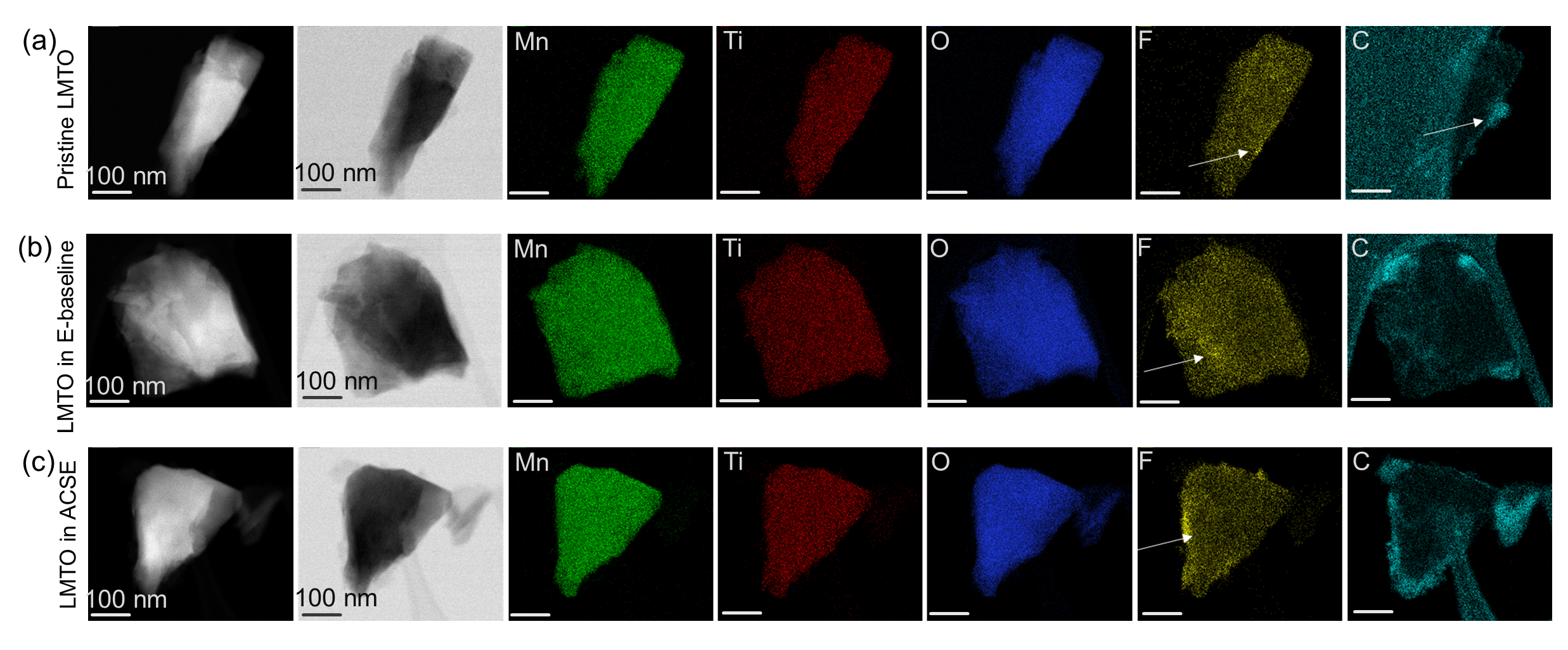


**Figure S13.** Elemental distribution in (a) pristine LMTO electrode particle, (b) cycled LMTO particle in E-baseline and (c) cycled LMTO particle in ACSE after 200 cycles.


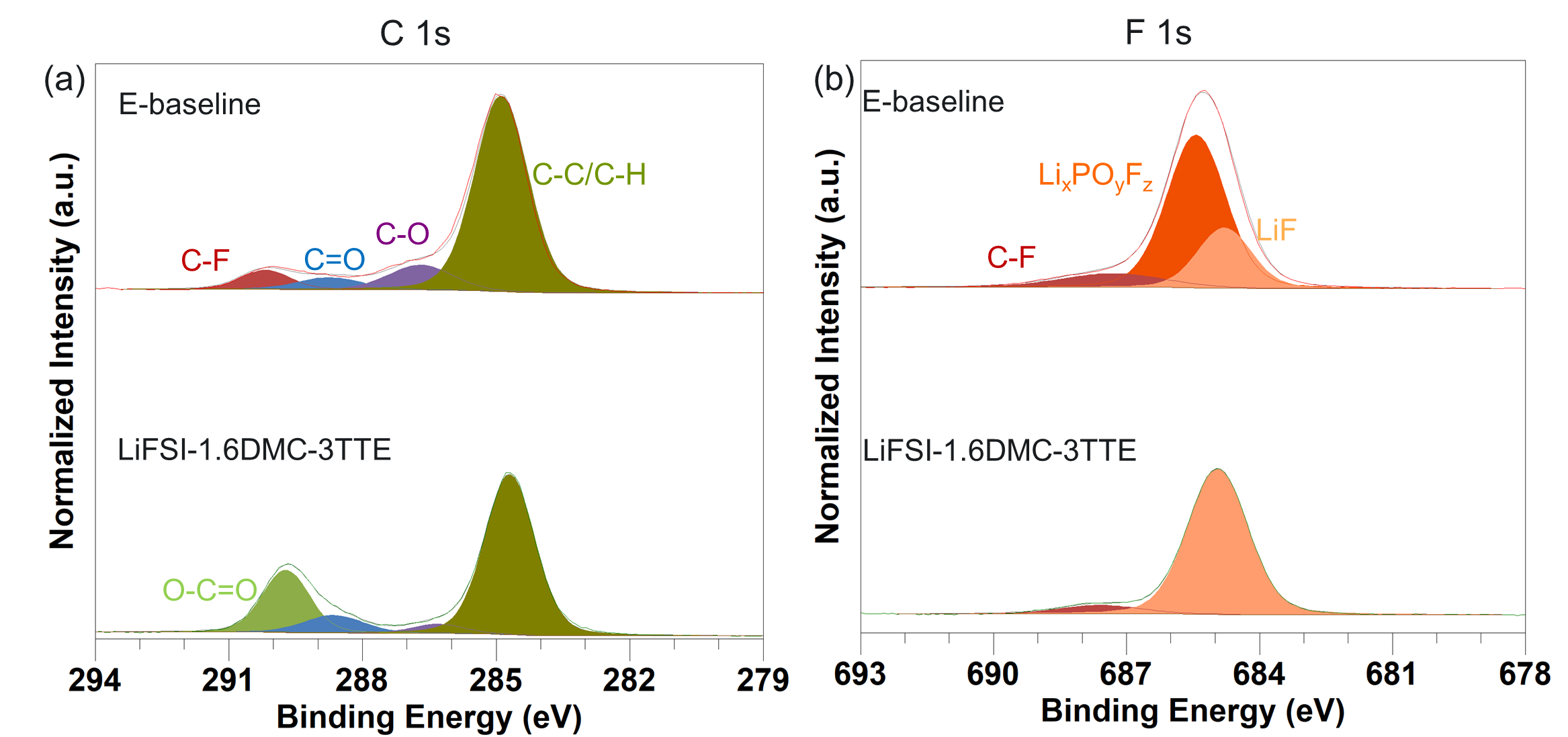


**Figure S14.** XPS spectra of (a) C 1s and (b) F 1s for the chemical compositions of the SEIs on Li metal anodes cycled with E-baseline and ACSE (LiFSI-1.6DMC-3TTE) collected at the 200th discharge state.

**References**

[1] a V. C. Wu, P. Zhong, J. Ong, E. Yoshida, A. Kwon, G. Ceder, R. J. Clement, *ACS Energy Lett.* **2024**, *9*, 3027-3035; b R. Giovine, E. Yoshida, V. C. Wu, Y. Ji, M. J. Crafton, B. D. McCloskey, R. J. Clement, *Chem. Mater.* **2024**, *36*, 3643-3654.
